# Mammals on the Margins: Identifying the Drivers and Limitations of Range Expansion

**DOI:** 10.1111/gcb.70222

**Published:** 2025-05-04

**Authors:** Alex J. Jensen, Benjamin R. Goldstein, Michael V. Cove, Krishna Pacifici, Elizabeth Kierepka, Brigit Rooney, William McShea, Roland Kays

**Affiliations:** ^1^ North Carolina Museum of Natural Sciences Raleigh North Carolina USA; ^2^ Department of Forestry and Environmental Resources North Carolina State University Raleigh North Carolina USA; ^3^ Smithsonian Conservation Biology Institute Front Royal Virginia USA

**Keywords:** area of habitat, citizen science, climate change, global change, iNaturalist, IUCN, North America

## Abstract

Accurately estimating species distributions is critical for tracking how biodiversity is shaped by global change. While some species are expanding their ranges, the importance of factors like climate change, habitat change, and human avoidance for explaining this expansion is not well understood. Here, we used observations of 94 North American mammals on iNaturalist to (1) identify errors of omission in the existing range maps; (2) differentiate between extra‐range populations that are likely products of natural expansions vs. introductions; and (3) test hypotheses about where natural range expansions occur. We found a substantial percentage of observations were outside both IUCN (16%) and Area of Habitat (36%) maps, suggesting that integrating contemporary citizen science data would improve existing range maps. We estimated that most observations outside IUCN ranges were natural expansions and 95% of species had at least one naturally expanding population. We also identified introductions for 36% of species, which were particularly extensive for several species. We show that natural range expansions are generally associated with a lighter human footprint and less habitat change and are not associated with warming temperatures. This suggests that habitat modifications by humans constrain the ability of species to expand their range to track a changing climate. We also found substantial variation in the directionality of effects from all factors across species, meaning that our species‐specific findings will be useful for conservation planning. Our study demonstrates that citizen science data can be useful for conservation by tracking how organisms are responding, or failing to respond, to global change.

## Graphical abstract

Using expert range maps and iNaturalist data, we show that the ranges of many North American mammals have expanded. Natural range expansions seem to be generally limited by human disturbance and not necessarily associated with climate change as we might expect.

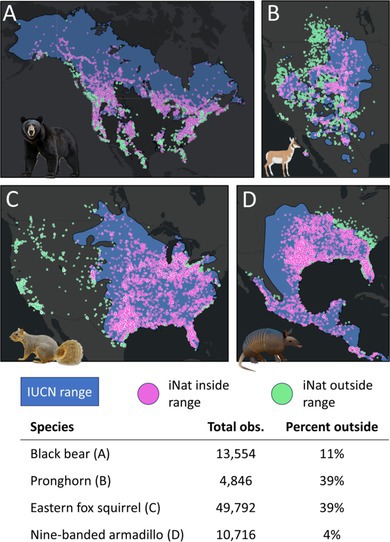

## 1 Introduction

Humans are causing rapid global environmental change, and understanding how other species are adapting is paramount for conservation. One of the fundamental ways in which species can respond to global change is by shifting their distributions to include areas that have become environmentally suitable (Lawing and Polly 2011; Poloczanska et al. 2013). In the context of warming temperatures from climate change, species are generally expected to shift their distributions to higher latitudes, higher elevations, and deeper depths (Rubenstein et al. 2023). However, there is mounting evidence that range shifts are not always directionally consistent with climate change—two recent reviews found consistency in only 47% and 59% of cases (Lawlor et al. 2024; Rubenstein et al. 2023). This high variability suggests that other factors are at least as important as climate, and some have suggested that human‐mediated habitat modification (e.g., urbanization and agriculture) could be limiting the ability of species to respond to climate change (Lenoir et al. 2020). However, animals respond in divergent ways to anthropogenic change (Blumstein et al. 2005; Fidino et al. 2021); thus, the same habitat change that restricts some sensitive species' capacity to shift could facilitate dispersal and colonization for others.

Tracking how animals respond to global change requires accurate and timely estimates of species distributions (Merow et al. 2017). Although distributions can be defined in various ways, we find the distinction between extent of occurrence (EOO) and area of occupancy (AOO) to be useful. EOO is defined as the area where a species is likely to occur and is often what is represented by range maps (Gaston and Fuller 2009). While multiple approaches exist to generate range maps, often the extent is manually defined based on occurrence data and expert opinion. Consequently, both EOO and range maps can be overly inclusive, such that unoccupied areas are erroneously included. AOO estimates are designed to reduce these errors of commission and are defined as the area actually occupied by the species (Gaston and Fuller 2009). One example of AOO estimates is area of habitat (AOH) maps, which is the suitable habitat available to a species within its range (Brooks et al. 2019). In theory, AOH maps should reduce errors of commission in range maps by removing unsuitable habitat within a given species' EOO. Regardless, both AOO and EOO are also subject to errors of omission (failing to include areas where species occur), which could happen if occurrence data are sparse or recent range expansions are not included in the range map.

One promising advance towards the goal of tracking range dynamics is the rise of citizen science platforms (e.g., eBird, iNaturalist), which contain millions of occurrence records and are growing exponentially (Kays et al. 2020). Because of their high spatiotemporal coverage and (in the case of iNaturalist) photo‐based sampling protocols, data from these platforms are particularly useful for identifying range expansions. For example, a global study of > 50,000 species' range maps quantified the percentage of occurrence data from the Global Biodiversity Information Facility (GBIF; a biodiversity data aggregator that includes citizen science data) outside each range and found a surprising percentage of observations outside range maps—25% to 46% on average across various taxa (Hughes et al. 2021). While many of these extra‐range observations likely represent legitimate range expansions, they could also be species misidentifications or locational errors in the citizen science data (Budde et al. 2017). Thus, demonstrating that extra‐range observations are reliable will be an important first step (Arbogast and Kerhoulas 2024; Marsh et al. 2024) towards testing hypotheses about the underlying drivers determining their locations.

Differentiating between extra‐range observations that represent natural range expansions (Barnes and Hoffman 2023) versus those that represent introductions by humans (Chapman et al. 2017) is also important for understanding how species are responding to global change. Although many studies have tested if range shifts are directionally consistent with climate change (Lawlor et al. 2024; Rubenstein et al. 2023), few have tested a larger suite of hypothesized drivers of natural range expansions (Table 1). If climate change does indeed lead to newly suitable environmental conditions for species to expand into, we predict that more extra‐range observations would occur where temperatures have recently warmed the most (Rubenstein et al. 2023). We also hypothesize that range expansion is generally more likely away from people (Haddad et al. 2015); therefore, we predict that extra‐range observations would be associated with a lighter human footprint (Venter et al. 2016) and with the presence of protected areas (Brennan et al. 2022). Alternatively, land use changes (e.g., deforestation or reforestation) could create newly suitable habitat for some species to colonize, though we expect these effects to be dependent on species‐specific habitat associations.

We tested these hypotheses using range maps and iNaturalist data from North American mammals. We also quantify the proportion of observations outside the existing range maps and develop a novel approach to differentiate between natural range expansions and introductions by humans. Ultimately, we demonstrate the utility of citizen science data for more accurately mapping contemporary species distributions and understanding where natural range expansions occur.

**TABLE 1:** Hypotheses, predictions, and variable metadata used to investigate the location of extra‐range observations of North American mammals. Plots of spatial data are available in Figure S2.

Hypothesis	Prediction (extra‐range observations associated with)	Spatial resolution	Data timeframe	Measure	Value range	Source
Climate is now suitable	Increased temperatures	4 km	1996–2000 to 2016–2020	Degrees C	−7.9 to 12	Thornton et al. (2022)
Habitat is now suitable	Forest height change	3 km	2000–2020	Meters	0–19	Potapov, Li, et al. (2021)
	Cropland change	3 km	2003–2019	Percent	0–72	Potapov, Turubanova, et al. (2021)
Human avoidance	Lower human footprint	1 km	2010	Intensity	0–50	Mu et al. (2022)
	Protected areas	Varies	2024	Protected or not	0 or 1	Protected Planet

## 2 Methods

### 2.1 Range Maps

Various organizations have created range maps, but the IUCN Red List maintains what is likely the most comprehensive and authoritative source (IUCN 2024). We downloaded extant‐only range maps from the IUCN Red List in August 2023. Extant‐only range maps exclude ‘extinct’ polygons and include polygons classified as a product of (re)introductions if they exist for a given species. For AOH maps, we used maps for the world's terrestrial mammals and birds by Lumbierres et al. (2022), where the authors removed areas from IUCN range maps based on each species' associations with habitat and elevation. These AOH maps were provided as 100 m × 100 m binary rasters. At least two studies have evaluated these AOH maps; one study used occurrence data from GBIF (Dahal et al. 2022) while the other used camera trap data (Chen et al. 2024). Both studies concluded that errors of omission were rare, yet more work is needed to formally evaluate AOH maps, especially relative to more inclusive range maps like from IUCN.

### 2.2 iNaturalist Data Curation

We batch‐exported data from iNaturalist.org in January 2023 by filtering for mammals in North America from January 1, 1900 to December 31, 2022. This initial dataset contained 1,857,235 observations, but we removed 14 duplicate observations with matching observation identification numbers, and all observations that were not research grade or were not identified to species. We further excluded observations of marine mammals except for polar bears (See Table S1 for scientific names); any based on sign, tracks, or bone because these types of observations could be more difficult to identify to species (Morin et al. 2016); any observations prior to the year 2000; and any with a positional accuracy (uncertainty) > 1 km because of our interest in accurately classifying observations as either within or outside ranges. Lastly, we excluded observations outside the United States, Mexico, and Canada because we were unable to find long‐term data on temperature outside of these countries. We retained 866,978 observations after applying these filters.

### 2.3 Inspecting Extra‐Range Observations

Given our focus on extra‐range observations, it was imperative to add additional confidence in the legitimacy of these observations before conducting analyses. To do this, we manually inspected extra‐range observations by uploading each species' iNaturalist observations and their IUCN range. We added a 5 km buffer to the range, which substantially reduced the number of extra‐range observations associated with small errors in the range maps (e.g., discrepancies between the range and coastlines). We then identified the observations that were outside this buffered range and inspected them by viewing them on iNaturalist. We inspected all solitary observations but in cases when there were hundreds of observations clustered together, we inspected enough to feel confident about the species' occurrence in the area. We excluded observations from further analyses for the following reasons: (1) species was misidentified; (2) based on a captive or non‐resident individual; (3) based on animal sign but not previously filtered; and (4) images were not of sufficient quality to identify the species. We only conducted this exercise for species (*n* = 94) that we could confidently differentiate from other non‐volant mammals based on confusion scores from (Kays et al. 2022); thus, we excluded bats and species that were rarely or never distinguishable from co‐occurring species (confusion scores of 3 or 4). After applying these filters to the dataset, we excluded five additional species with no remaining iNaturalist observations. We also excluded six additional species because the precise location of more than 75% of their observations was obscured (often automatically by iNaturalist because they were species of conservation concern; e.g., black‐footed ferret). This left 83 species.

### 2.4 Objective One: Quantifying Extra‐Range Observations

Our objective was to quantify the percentage of observations that fall outside IUCN and AOH range maps. Using our cleaned dataset of 83 species, we calculated the percentage of observations which were outside each distribution. Given the AOH rasters were finer scale than our positional accuracy filter (< 1 km), we also separately calculated the percentage of extra‐range observations after excluding observations with > 100 m and > 50 m positional accuracies. We found that the average difference in percentage when comparing 1 km with 100 m and 50 m was < 1% across species, suggesting that a mismatch between the scale of the raster and positional accuracy did not bias our estimates. The AOH raster did not include a distribution for red fox in North America, so we excluded this species from any summaries of the AOH data.

### 2.5 Objective Two: Classifying Observations by Means of Arrival

We used two strategies to differentiate between extra‐range observations likely to represent natural range expansions versus introductions by humans. First, we determined that observations relatively far from the IUCN range map and far from other observations that appeared to be connected to those close observations were likely to represent introductions or isolated vagrant individuals (hereafter collectively referred to as introduced). To identify those observations, we created a 50 km buffer around each extra‐range observation and dissolved these polygons into each other and the range (Figure S1). This extended the original range map in some directions by observations within 50 km of the range map or each other. However, in some cases, there were polygons that were not connected to the original range map; we classified all observations within these polygons as introduced.

The second strategy we used was information about the means of establishment from the observation's metadata. Users on iNaturalist are able to flag observations as ‘introduced’ if they have information that the population arrived to the area via anthropogenic means. The iNaturalist community has also curated regions of known introductions for many mammal species, in which new observations are automatically flagged as introduced (e.g., eastern gray squirrels in California). We exported North American mammal observations with this flag using the iNaturalist export tool on August 15, 2024 and identified those observations in our dataset. Thus, for each species, we classified observations as introduced if they were (a) within polygons not connected to the original range map or (b) flagged as introduced on iNaturalist. We then excluded observations which were within 1 km of each species' range map (functionally treating them as intra‐range) because there were minor spatial discrepancies between range maps and the coastline, which this buffer resolved. All remaining observations we classified as likely to represent natural range expansions. We excluded species from this analysis if we identified < 30 total extra‐range observations, following sample size recommendations from the resource selection literature (Millspaugh et al. 2006).

We also estimated the number of populations that represented natural range expansions and introductions for each species. For natural range expansions, we counted the number of polygons that were contiguous with the IUCN range, whereas we counted the polygons not contiguous with the IUCN range for introductions. In both cases, we excluded polygons smaller than 2× the area of a single 50 km radius polygon, in order to minimize counting polygons that represented vagrant individuals.

### 2.6 Objective Three: What Explains the Location of Natural Range Expansions?

Here, we tested a suite of hypotheses (Table 1; Figure S2) to explain the location of extra‐range observations that represent natural range expansion. Our first hypothesis was that warming temperatures would facilitate range expansion, so we calculated the change in temperature in recent decades. We downloaded 1 km × 1 km rasters of the average maximum temperature from 1996 through 2000 and 2016–2020 from the NASA DAYMET database (Thornton et al. 2022). We then averaged values in each cell across each 5‐year period and subtracted the older period from the more recent period. Thus, a positive number in a raster cell would indicate an increase in maximum temperature.

Second, we hypothesized that habitat change would facilitate range expansion, so we used data on forest height change and cropland change in recent decades. The forest height change dataset was from (Potapov, Li, et al. 2021), where the authors provided 30 m × 30 m rasters of forest height gain and forest height loss in meters from 2000 to 2020 (Table 1). We resampled these rasters to 3 km × 3 km to match the cropland change dataset, which was from (Potapov, Turubanova, et al. 2021) and provided as percent gain or loss from 2003 to 2019.

For our hypothesis that animals generally avoid humans, we used a human footprint index and a database of protected areas. For human footprint, we used a 1 km × 1 km raster from (Mu et al. 2022) from 2010, which was roughly in the middle of the habitat change and temperature datasets. Human footprint is a composite index, combining human population density, land use, and infrastructure (e.g., built‐up areas, nighttime lights), and human access (e.g., roads, railways). For protected areas, we downloaded the Protected Planet database (UNEP and IUCN 2024) in May 2024, which contained polygons for approximately 72,000 protected areas from across North and South America.

We retrieved the value of each covariate at the location of every observation in our dataset. Some spatial data did not exist at some coordinate locations, so we replaced NAs using several methods. Some data were missing because small (< 1 km) positioning errors in the location caused the observation to be erroneously placed in water, which we could confirm by looking at the observation and seeing that the photo was taken somewhere nearby on land. This occurred with the human footprint and temperature change data (*n* = 4369 and 1175 observations, respectively), which we resolved by replacing each NA with the average value from the 10 nearest raster cells. A small number of observations (< 30) were also missing forest and cropland data, but these were all in northern Canada and northern Alaska (where neither of these land cover types exist), so we replaced the NAs in this case with zero, signifying no change.

We retrieved the value of each covariate at the location of every observation in our dataset. Some spatial data did not exist at some coordinate locations, so we replaced NAs using several methods. Some data was missing because the observation was in the water, which was often due to small (< 1 km) positioning errors in the location, which we could confirm by looking at the observation and seeing that the photo was taken on land. This occurred with the human footprint and temperature change data (*n* = 4369 and 1175 observations respectively), which we resolved by replacing each NA with the average value from the 10 nearest values. A small number of observations (< 30) were also missing forest and cropland data, but these were all in northern Canada and northern Alaska (where neither of these land cover types exist), so we replaced the NAs in this case with zero, signifying no change.

**FIGURE 1:**
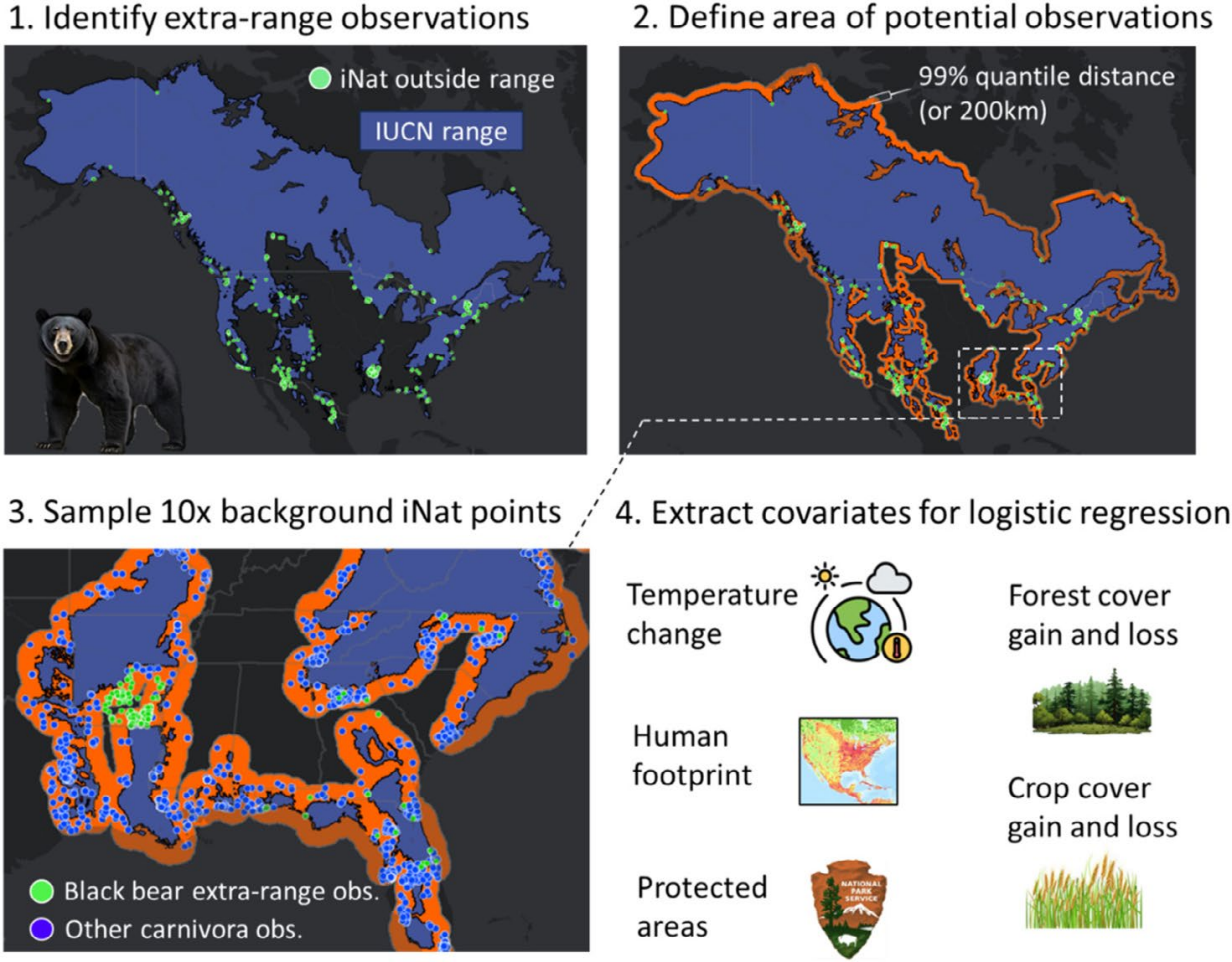
Workflow for how we tested hypotheses related to the location of extra‐range observations for North American mammals using black bear as an example. In panel 1, we show the distribution of iNaturalist black bear observations overlayed on their IUCN range. In panel 2, we focus on extra‐range observations and show how we identified the area outside the range where extra‐range observations could have occurred. In panel 3, we zoom in on the southeastern portion of panel 2 and show how we used observations of other species in a given species' order (e.g., Carnivora) as background points. In panel 4, we show the environmental variables we used to test our hypotheses. Map lines delineate study areas and do not necessarily depict accepted national boundaries.

To test our hypotheses, we compared data from the location of extra‐range observations to locations outside of each species' range where they could have occurred (Figure 1). While it is likely that some extra‐range observations do not represent expansions, but rather established populations erroneously excluded from the IUCN range, we expect these errors to be random in relation to the environmental data representing our hypotheses. Thus, our null hypothesis was that extra‐range observations representing natural range expansion could occur anywhere near the range (on land). To appropriately represent these areas of possible expansion, we identified a species‐specific region from which we could draw background observations (Figure 1). We measured the distance from the 1 km extended IUCN range map to each extra‐range observation and calculated the distance that captured 99% of extra‐range points (99% quantile). We buffered the extended range map by this distance and defined this buffered area as the region where extra‐range observations could have occurred (Figure 1). We excluded observations outside these species‐specific buffers, then excluded species with < 30 extra‐range observations remaining, leaving 44 species.

We then generated a set of species‐specific background observations within this buffered region. To represent spatial heterogeneity in iNaturalist sampling effort, we drew background comparison locations from other iNaturalist observations from within the species' order (Figure 1). We grouped nine‐banded armadillos and Virginia opossum with carnivores because these two species did not have enough observations of other species within their order, so we included them with carnivores as they are generally similarly sized (and therefore presumably have similar detectability). We randomly selected up to 10× the number of extra‐range observation points as background points. There were five species for which there were not 10× points available (though three had > 5×), in which cases we used all observations of species in the target order as background.

We used a Bayesian hierarchical model to compare the spatial data extracted from the extra‐range observations with background observations. Our response variable was whether a given observation was our focal species or a background observation. Our predictor variables were the spatial data we extracted at each observation (Table 1). We scaled and centered the continuous predictor variables, checked for collinearity by calculating all pairwise Pearson's correlation coefficients and found no significant correlations (all values were < 0.25). We then fit a hierarchical model, where information‐sharing across species allowed the effects of our predictor variables on individual species to be informed by the community mean values (drawing estimates from species with relatively few observations towards the community mean). Specifically, we fit a multispecies logistic regression mixed effects model where for each species (*j*), we estimated the effect of our environmental variables on the probability of an observation (*i*) being the focal species (i.e., the datum *y*_*ij*_ = 1) or a background observation (*y*_*ij*_ = 0):

$$y_{\mathrm{ij}}\sim \text{Bernoulli}\left(p_{\mathrm{ij}}\right)$$

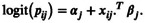

where *x*_*ij*_ is a vector of covariate data for the *i*th observation associated with species *j*, and *β*_*j*_ is a vector of coefficients giving the effect of those covariates on the logit probability. Under this model, which is a multispecies extension of a generalized linear mixed effects model (Bolker et al. 2009), the probability that an observation just outside the range of species *j* is an observation of species *j* has a baseline of $\text{logi}t^{-1}\left(a_{j}\right)$ when all covariates are at their mean values and may change on a logit‐linear scale according to the covariates *x*_*ij*_ with effects *β*_*j*_. This model is also closely related to similar presence‐background used to model species' distributions from presence‐only data (Elith et al. 2006; Pearce and Boyce 2006), using observations of non‐target species to represent the distribution of sampling effort in covariate space.

To share information across species, we used random coefficients and assumed that the effect of each covariate *k* on each species *j* was a random effect drawn from a shared normal distribution, as

$$\beta_{\mathrm{jk}}\sim N\left(\mu_{k},{\sigma_{k}}^{2}\right).$$

This method of sharing information among a community of species with similar life histories is commonly used in ecology, such as in the multispecies occupancy model (Iknayan et al. 2014). The species‐specific intercepts, *α*_*j*_, interpreted as the probability that a point is an observation of the focal species when all covariates are at their mean values, are estimated as fixed effects as we do not consider these to be drawn from a common distribution as we do not expect that species should necessarily have similar baseline detection rates in iNaturalist. We specified a normal prior distribution with mean = 0 and standard deviation = 5 for each *μ*_*k*_ and *α*_*j*_, as well as a uniform prior standard deviation with bounds = (0.01, 10) for each standard deviation *σ*_*k*_. We used the nimble package (De Valpine et al. 2017) to estimate our model with Markov chain Monte Carlo (MCMC) using 3 chains of 20,000 iterations each with a 1000 sample burn‐in period. We confirmed that MCMC sampling had converged by checking R‐hat values for each species‐covariate combination and found that all values were ≤ 1.1 (Gelman and Rubin 1992). We provide our code and data for this model here: https://figshare.com/s/f46608c39624b5f2827d.

## 3 Results

**FIGURE 2:**
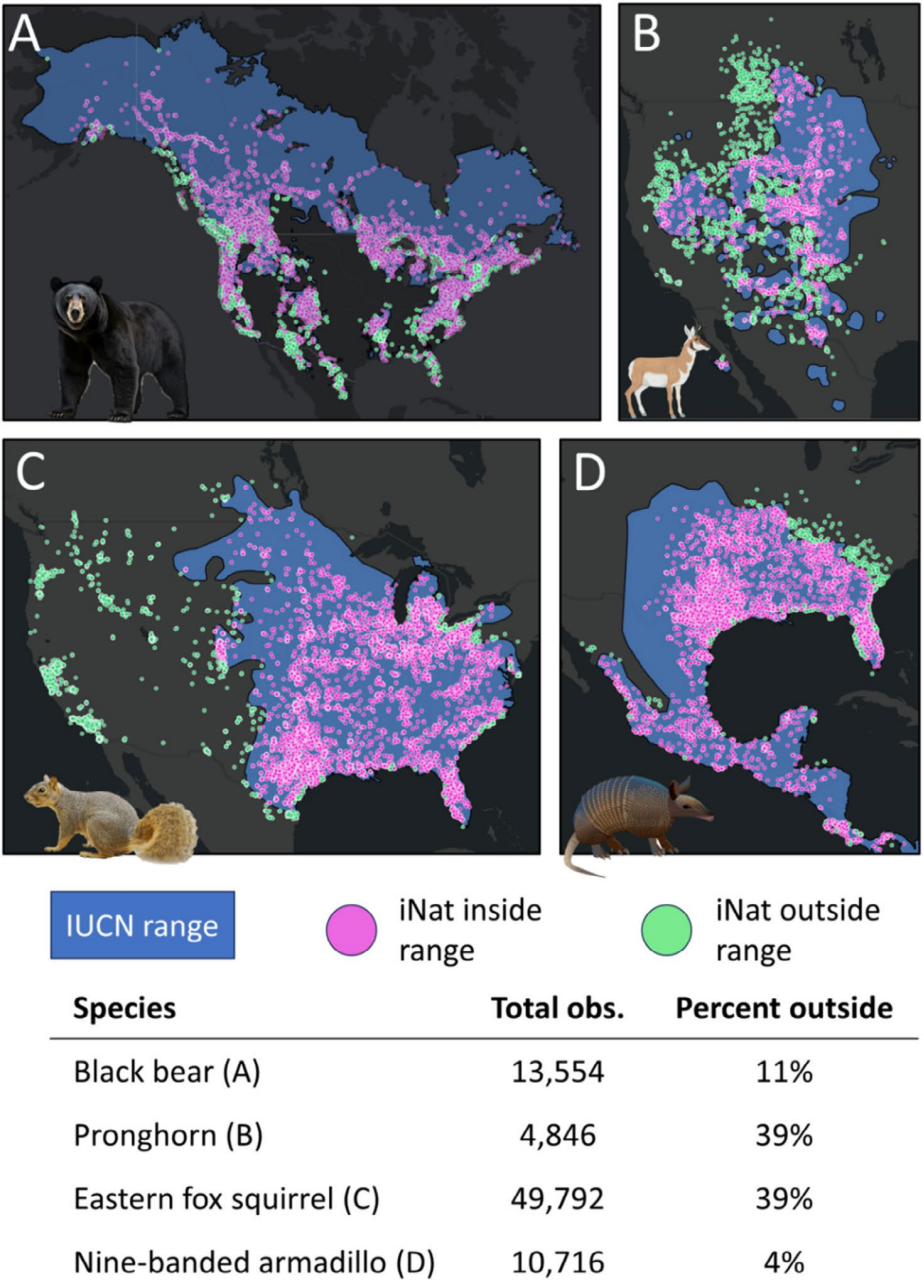
Four species that illustrate variation in how well IUCN range maps capture the distribution of iNaturalist observations in North America. In A, we show that black bear extra‐range observations are in many places, but not very far from the edge of the range. In B, we show a striking amount of observations outside of pronghorn range. In C, we show that the introduction of the eastern fox squirrel to many western cities has not been captured by the IUCN range. In D, we show how iNaturalist observations are capturing the continued range expansion of the nine‐banded armadillo, and how the northwestern edge of their range is likely too extensive. Map lines delineate study areas and do not necessarily depict accepted national boundaries.

We investigated 59,622 iNaturalist observations outside the ranges of 94 North American mammal species: 41 rodents, 29 carnivores, 10 rabbits, 10 ungulates, 2 shrews, 1 opossum, and 1 armadillo (Figure S3). We found that the vast majority of these extra‐range observations (98%) were trustworthy. Of the 2% we rejected, 75% of these were rejected because they were sign (i.e., tracks, scat, and bone), while the remainder were rejected for the various other reasons we list in the Methods section. We rejected a disproportionate percentage of observations (29%—62%) for several carnivore species (brown bear, gray wolf, arctic fox, and puma) because sign observations were common for these species, and there were relatively few total extra‐range observations. We also noted that 72 species (77%) had large areas within their range without iNaturalist observations; those in remote Canada are likely explained by a lack of iNaturalist effort (Figure 2A), but other areas have extensive observations of other species and thus likely represent range maps that are too extensive (e.g., the western edge of the nine‐banded armadillo's range; Figure 2D).

### 3.1 Objective One: Quantifying Extra‐Range Observations

**FIGURE 3:**
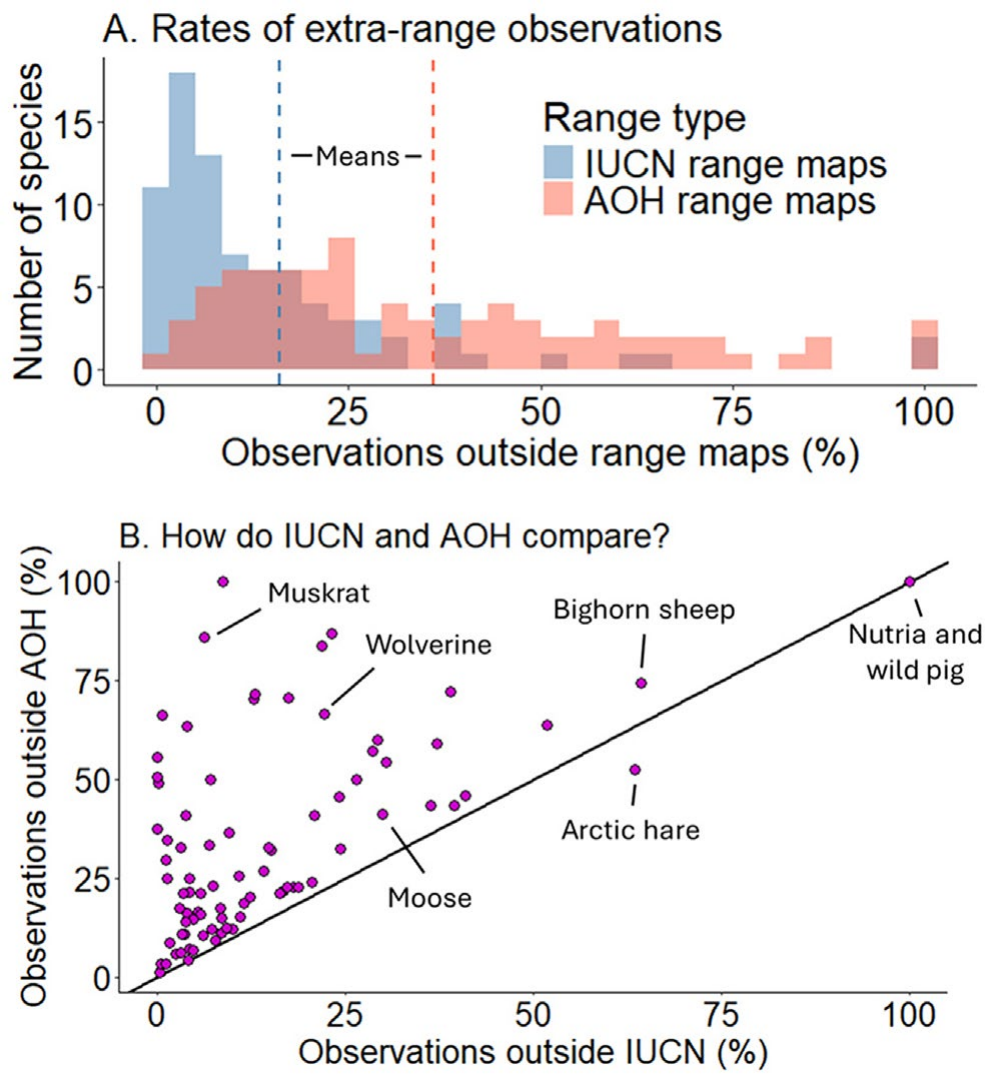
Visual summaries of how well IUCN ranges and Area of Habitat (AOH) maps capture iNaturalist observations of 83 North American mammals. In A, we show the distribution of the percentage of extra‐range observations, with the dotted lines representing the mean values for each range type. In B, we show the percentage of extra‐range observations for each species, where most species have a larger percentage outside their AOH map compared to their IUCN range.

Using 513,463 observations from 83 species, we found that the average percentage of extra‐range observations across species was 16% for IUCN ranges and 36% for AOH maps (Figure 3; Table S1). Three species had zero observations outside of their IUCN range, but these species either had very few total observations (round‐tailed muskrat, *n* = 8; Coues's rice rat, *n* = 9) or lived in regions without much iNaturalist effort (polar bear; *n* = 212). All observations for wild pigs and nutria were outside their IUCN ranges and AOH maps because these ranges did not include their North American distributions. Several other species had more than 50% of their observations outside IUCN ranges, including bighorn sheep (64%) and mountain goat (52%).

### 3.2 Objective Two: Classifying Observations by Means of Arrival

**FIGURE 4:**
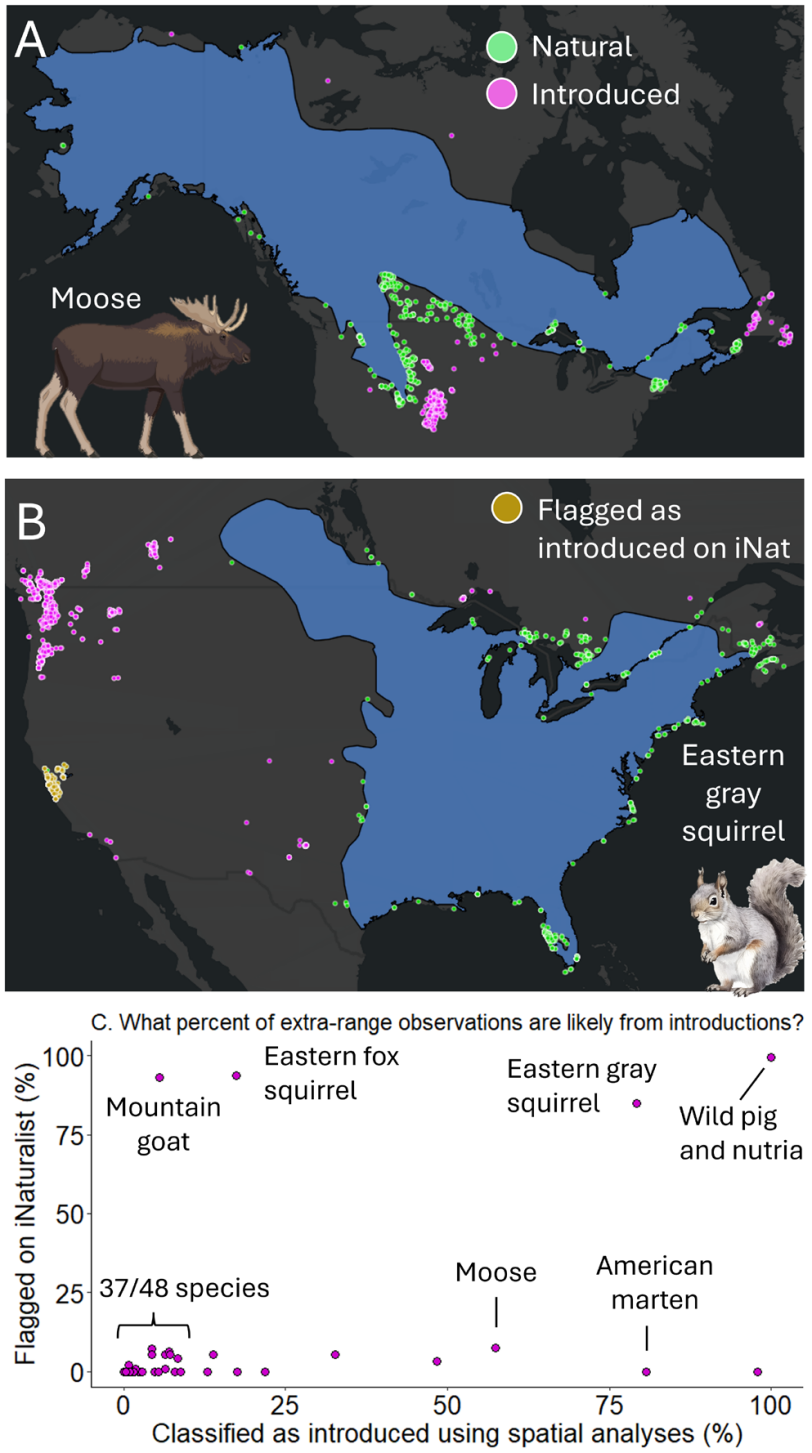
We classified extra‐range observations as likely to either represent (1) natural range expansions or (2) introductions by humans and vagrant individuals. We show two example species in the top panels—moose (A) and eastern gray squirrel (B), and maps for all species in Data S1. We only show observations flagged as introduced on iNaturalist if they are not also classified as introduced by our spatial analyses. In C, we show the percentage of extra‐range observations classified as introduced for 83 species. Map lines delineate study areas and do not necessarily depict accepted national boundaries.

We classified extra‐range observations for 48 species and found that natural range expansions typically represented the majority (84% on average) of extra‐range observations across species (Figure 4C). Yet introductions identified via our spatial analysis represented the majority of extra‐range observations for six species, including moose (57%), eastern gray squirrel (79%), and American marten (81%; Figure 4). Most species (30/48) did not have any extra‐range observations flagged as introduced by iNaturalist, but there were five species with more than 50% of their observations flagged (Figure 4C), and eight species with flagged observations which we would have otherwise classified as natural expansions (Figure 4B,C).

We estimated the number of independent natural range expansions for each species and found that 95% of species had at least one population with an average of eight (range = 1–26) across species. We identified fewer species (36%) with introductions, and there was an average of one (range = 1–8) across species.

### 3.3 Objective Three: What Explains the Location of Extra‐Range Observations?

**FIGURE 5:**
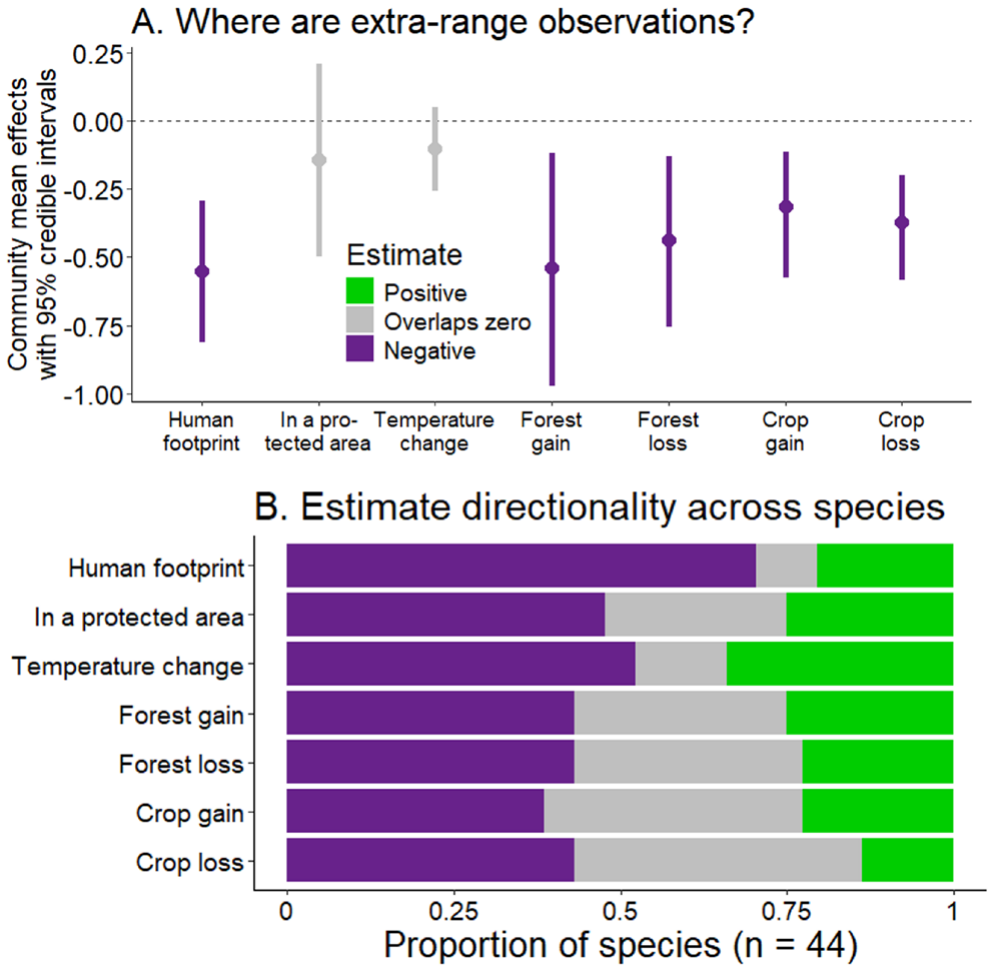
Results from our analysis on where extra‐range observations likely representing natural range expansions are for North American mammals. In A, we show the estimated community mean effects. In B, we show the proportion of species‐specific random slope estimates which were positive, overlapped zero, or negative (based on 95% credible intervals). Although most of our variables have a significant effect on the mean community response (A), we observe a substantial amount of variation among species (B).

All 44 species had significant environmental factors explaining where they were naturally expanding (Data S1), but these were quite variable across species (Figure 5). By sharing information in our community model, we found support for our human avoidance hypothesis, where extra‐range observations tended to be in areas with a lighter human footprint (estimated mean effect [95% credible intervals] = −0.55 [−0.81: −0.29]). Indeed, human footprint had a significant negative effect on 70% (*n* = 31) of species, which was the most consistent variable effect across species (Figure 5). This suggests that dispersal is generally limited by human footprint.

We did not find support for our human avoidance hypothesis in the context of protected areas, which had a neutral effect across species (−0.14 [−0.50: 0.21]). Yet we also found high variability in the species‐specific effects from protected areas, which were significantly positive or negative for 75% of species (Figure 5). The strongest positive effects were for fisher (3.30 [2.52: 4.18]), mountain goat (1.85 [1.36: 2.36]), and golden‐mantled ground squirrel (1.74 [1.04: 2.51]), suggesting that protected areas facilitate dispersal for these species in particular. The strongest negative effects were for puma (−2.47 [−3.80: −1.38]), 13‐lined ground squirrel (−2.23 [−3.16: −1.42]), and red fox (−2.00 [−2.84: −1.24]).

We did not find support for our hypothesis that habitat change would generally facilitate range expansion. Instead, we found that extra‐range observations tended to be in areas with less habitat change across our four different measures: forest loss (−0.43 [−0.75: −0.13]), forest gain (−0.54 [−0.97: −0.12]), crop loss (−0.37 [−0.58: −0.20]), and crop gain (−0.31 [−0.57: −0.11]). However, there were two species (Bighorn sheep and Douglas' squirrel) for which all four had negative effects. The majority (52%) of species had at least one negative and one positive relationship to these four variables, suggesting that habitat change (particularly forest gain, forest loss, and crop gain; Figure 5) facilitates natural range expansion in species‐specific ways.

Temperature change had a neutral effect on the location of extra‐range observations across species (−0.10 [−0.26: 0.05]), although its effects were significantly negative or positive for 86% of individual species (Figure 5). Negative effects were strongest for red fox (−1.34 [−1.70: −0.99]), gray fox (−0.89 [−1.08: −0.69]), and white‐nosed coati (−0.86 [−1.14: −0.58]), suggesting that these species are colonizing areas that have warmed less. Temperature change had a positive effect on 15 species, including rock squirrel (1.12 [1.01: 1.23]), golden‐mantled ground squirrel (0.83 [0.44: 1.23]), and western gray squirrel (0.60 [0.31: 0.76]).

## 4 Discussion

Accurately mapping and tracking changes in species distributions is critical for understanding how organisms are responding to global change (Poloczanska et al. 2013). Here, we used vetted occurrence data from iNaturalist to quantify errors of omission in existing range maps for North American mammals and test hypotheses concerning where natural range expansions have occurred. We found that a substantial proportion of observations were outside existing range maps (16% for IUCN and 36% for AOH on average), suggesting that there is an opportunity to better integrate iNaturalist data into revisions of mammal range maps. Using a rapidly growing set of photo‐vouchered occurrence data and a novel approach to identify means of arrival, we were able to document natural range expansions for 95% of species, as well as identify introduced populations for 36% of species. We found substantial variation in the factors predicting where natural range extensions occur across species, but on average, these were more likely to occur in areas with a lighter human footprint and less habitat change. These findings suggest that human‐driven habitat disturbance generally limits the ability of species to respond to a changing climate (Lenoir et al. 2020).

Human footprint had the strongest and most consistent (negative) effect on range expansion. This finding is generally consistent with past research showing that heavier human impacts on the landscape lead to reduced dispersal ability (Correa Ayram et al. 2017; Hand et al. 2014), more range contractions (Yackulic et al. 2011), and that they better predict range size than biological traits (e.g., body size, trophic level; 35). We found that human footprint had a negative effect even for two squirrels that were introduced to cities across western North America (eastern gray squirrel and eastern fox squirrel). One potential explanation for this counterintuitive finding is that these species naturally colonize habitat with minimal human disturbance faster than disturbed habitat, despite doing well once introduced in urban areas. More broadly, this human footprint finding is concerning in the context of limiting the ability of species to respond to climate change. In particular, if species are not able to track shifting temperatures due to human disturbance and fragmentation limiting their dispersal, their distributions could shrink (Lenoir et al. 2020). Indeed, this is one potential explanation for why we and other studies did not find that range expansions were generally associated with warming temperatures (Lawlor et al. 2024; Rubenstein et al. 2023).

Although temperature change had a neutral effect overall, we identified many species‐specific relationships to temperature change that will be useful for conservation planning (Data S1). For example, in line with our predictions, 15 species seemed to be naturally expanding in areas that have warmed more. Several squirrel species (eastern gray squirrel, eastern chipmunk, and 13‐lined ground squirrel), in particular, clearly had the majority of their extra‐range observations beyond the northern limits of their range, suggesting that the ranges of these species may be expanding into regions that were previously too cold. Indeed, although this pattern was not apparent for western gray squirrel using our data, Sultaire et al. (2024) suggest that this species is shifting its range as expected in response to climate change. Perhaps sciurids are particularly well adapted to respond to climate compared to other mammalian taxa. In contrast, 23 species have expanded more in areas that have warmed less (or even cooled) in recent decades. This makes sense for some of these species who range widely in northern latitudes, like red fox, porcupine, black bear, snowshoe hare, dall sheep, common muskrat, beaver, and caribou, but not for all 23. The remaining species (including those with neutral effects from temperature change) could be constrained in their ability to track climate change and therefore most in need of conservation interventions like assisted migration (Twardek et al. 2023). However, part of the variation we found could also be a function of species‐specific responses to climate change, especially given species traits (e.g., body size, movement ability) have been found to be weak predictors of range shifts (Estrada et al. 2016; MacLean and Beissinger 2017).

Another key finding from our study was that habitat change—the loss or gain of forests and cropland—constrained range expansion for a substantial percentage of species (33%—45%). This result was surprising given that we hypothesized habitat change would open new suitable habitats for potential expansion. Instead, this suggests that habitat stability likely promotes range expansion regardless of whether that habitat is natural (forests) or anthropogenic (cropland). Given these findings, it is possible that managed forests (i.e., those periodically cleared for timber) are less conducive to range expansions relative to unmanaged forests (Paillet et al. 2010); though, we hypothesize that management would primarily limit establishment (e.g., through lower den availability) and not necessarily dispersal. If stability does indeed facilitate range expansion, protected areas should likewise facilitate range expansion, given they are generally more protected from human disturbance (Nagendra 2008). Although protected areas did have a positive effect on 31% of species, they had a neutral effect overall—perhaps because they are not actually as buffered from habitat change as we might think (Guerra et al. 2019) or because they do not facilitate dispersal more than non‐protected areas (Parks et al. 2020). Yet we also found high variability in the effects from habitat change both across and within species, including having positive effects for 11%—31% of species. Taken together, these findings suggest that disturbance generally constrains range expansion, but there is substantial variability in how different species respond (Blumstein et al. 2005; Fidino et al. 2021).

Although natural range expansion seems to be a key reason why extra‐range observations exist (Barnes and Hoffman 2023), we also show that introductions are an important driver of range expansions for some species. For example, both eastern gray squirrels and eastern fox squirrels have been introduced into the urban west, and now at least 6 and 8 separate populations of these species persist and are likely continuing to spread. Several species were also introduced on islands in the early–mid 1900s, including moose, mink, American red squirrel, and eastern chipmunk on Newfoundland, but these populations are not included in their IUCN range maps. Similarly, North America is not included in IUCN range maps for wild pig or nutria despite multiple introductions to North America over a century ago. These results suggest that range maps could improve by including all known areas containing (re)introduced populations (Wallach et al. 2020). More broadly, our findings show that humans facilitate range expansions for many species, and that citizen science data can help track where this is occurring.

Our findings suggest that existing range maps could improve in several other ways as well. First, if not used already, range maps should use citizen science data to help identify where species occur, especially given we found that the vast majority of extra‐range observations are legitimate. It should be relatively straightforward to overlay these data on existing range maps and extend the maps where appropriate. This could likely be done in several ways, including buffering the extra‐range observations and dissolving those buffers with each other and the map, or creating convex hulls with the extra‐range observations. Second, we show that efforts to remove unsuitable habitat from range maps (i.e., AOH maps) may be removing too much area, as we found that AOH maps had more than double (36%) the extra‐range observations compared to IUCN ranges (16%) on average. This suggests that researchers should be cautious about adopting AOH maps as a superior alternative to traditional range maps, at least as presently implemented. Lastly, given the rapid pace of global change, we support previous calls for more frequent updates to IUCN range maps (Juffe‐Bignoli et al. 2016; Rondinini et al. 2014). A large percentage (38%) of IUCN range maps in our dataset were last updated in 2008, while another 34% were updated in 2016 (Table S1). This suggests that there should have been another push around 2024 but it remains to be seen if the latest maps will incorporate iNaturalist observations. We envision a future where range maps are frequently and automatically updated as new occurrence data become available (Suárez‐Castro et al. 2024), perhaps facilitated by platforms such as sRedList (Cazalis et al. 2024) or Map of Life (Marsh et al. 2024).

There are also several areas where future work could further increase the utility of citizen science data for informing species distributions. For example, a better understanding of how spatial variation in iNaturalist effort influences where we detect range expansions is needed, including how that effort is shaped by socioeconomics (Chapman et al. 2024). For example, perhaps some species are expanding their range within Canada, but this is not captured because there is little iNaturalist effort in this region. It would also be interesting to be able to classify range expansions as either recovery of previously lost range or the colonization of entirely new areas. This would require spatial data on historical ranges (e.g., Pacifici et al. 2019). Compared to range expansion, a more complicated problem is figuring out how to use citizen science data to subtract areas of the range which represent range contractions or errors of commission. This is tricky because a lack of occurrence data could be a function of lack of occurrence, little search effort, or species traits that make detection difficult. However, perhaps some sort of spatial process that accounts for effort and uncertainty could work, like the approach developed by Suárez‐Castro et al. (2024). Future studies might also test additional hypotheses, which we did not include in our analyses. For example, topography could partially explain where natural range expansions occur, as mountains or major rivers could serve as barriers to dispersal (Jensen et al. 2024). Likewise, changes in precipitation patterns could have subtle effects on habitat suitability that might not be captured by changes in land cover (Tingley et al. 2009). Regardless, we encourage future studies to interrogate the full suite of factors that could influence range dynamics, not just climate.

Species distributions are commonly used in a variety of important contexts including prioritizing regions for conservation funding (Brum et al. 2017; Maxwell et al. 2020), identifying key biodiversity areas (Plumptre et al. 2024), mapping threats to terrestrial vertebrates (Harfoot et al. 2021), and tracking invasions (Lyons et al. 2020). Thus, finding ways to estimate distributions accurately is important for tracking how species are responding to global change (Poloczanska et al. 2013). We show that humans are directly responsible for some range expansions, although the majority seem to be a product of natural dispersal and colonization from range edges. Tracking these natural range expansions reveals that some species are adjusting to our changing planet, but the dispersal of others is constrained by human disturbance.

## Author Contributions

**Alex J. Jensen:** conceptualization, data curation, formal analysis, investigation, methodology, visualization, writing – original draft. **Benjamin R. Goldstein:** formal analysis, methodology, validation, writing – review and editing. **Michael V. Cove:** conceptualization, funding acquisition, methodology, supervision, writing – review and editing. **Krishna Pacifici:** formal analysis, funding acquisition, methodology, project administration, supervision, writing – review and editing. **Elizabeth Kierepka:** project administration, supervision, writing – review and editing. **Brigit Rooney:** methodology, writing – review and editing. **William McShea:** conceptualization, funding acquisition, methodology, project administration, supervision, writing – review and editing. **Roland Kays:** conceptualization, funding acquisition, investigation, methodology, project administration, supervision, writing – review and editing.

## Conflicts of Interest

The authors declare no conflicts of interest.

## Supporting information


Data S1.

## Data Availability

The data that support the findings of this study are openly available in Figshare at https://figshare.com/s/f46608c39624b5f2827d and https://figshare.com/s/e154a5dbaf79ffc7e42d. IUCN range maps were obtained from the IUCN red list at https://www.iucnredlist.org. Area of Habitat maps were obtained from Dryad at https://doi.org/10.5061/dryad.02v6wwq48. Forest and Crop gain and loss spatial data was obtained from the Global Land Analysis and Discovery group at https://glad.umd.edu/dataset/GLCLUC2020/. Climate data were obtained from ORNL DAAC at https://doi.org/10.3334/ORNLDAAC/2130. Protected areas were obtained from Protected Planet at www.protectedplanet.net. Human footprint data were obtained from Figshare at https://doi.org/10.6084/m9.figshare.16571064.
