# Supplementary material for: Mammals on the Margins: Identifying the Drivers and Limitations of Range Expansion
Source: Glob Chang Biol. 2025 May 4;31(5):e70222. doi: 10.1111/gcb.70222 (PMC12050905; doi:10.1111/gcb.70222)
Supplement: Supplementary file 1 — Data S1. [file GCB-31-e70222-s001.docx]

**Mammals on the margins: identifying the drivers and limitations of range expansion**

**SI Appendix**

**
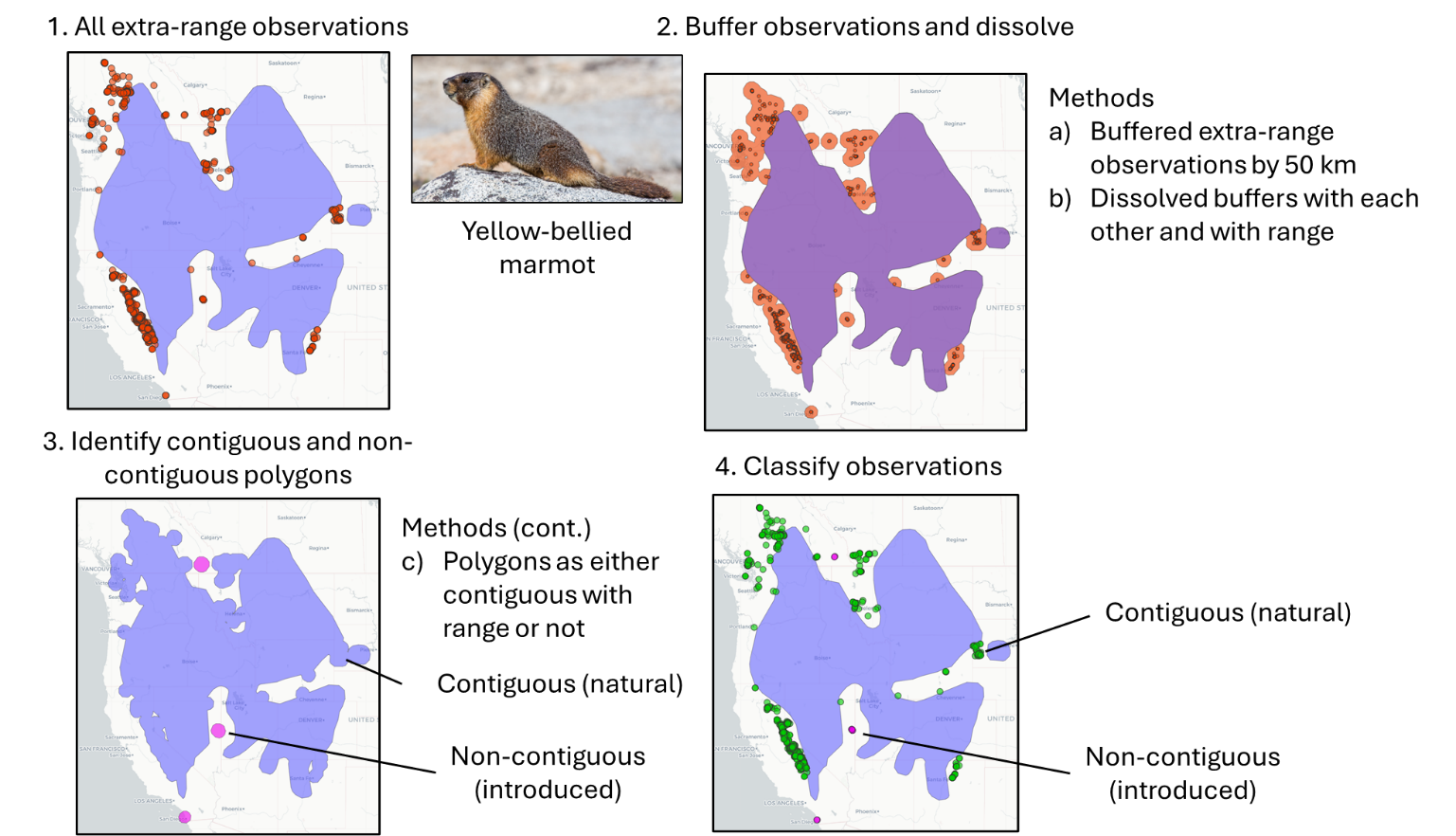
**

**Fig. S1.** Example workflow for how we classified observations as likely to either be a product of natural range expansion vs. an introduction by humans or vagrant individual. We also classified observations as introduced if they were annotated as such within iNaturalist (not shown here but in Figure 4). Map lines delineate study areas and do not necessarily depict accepted national boundaries.

**
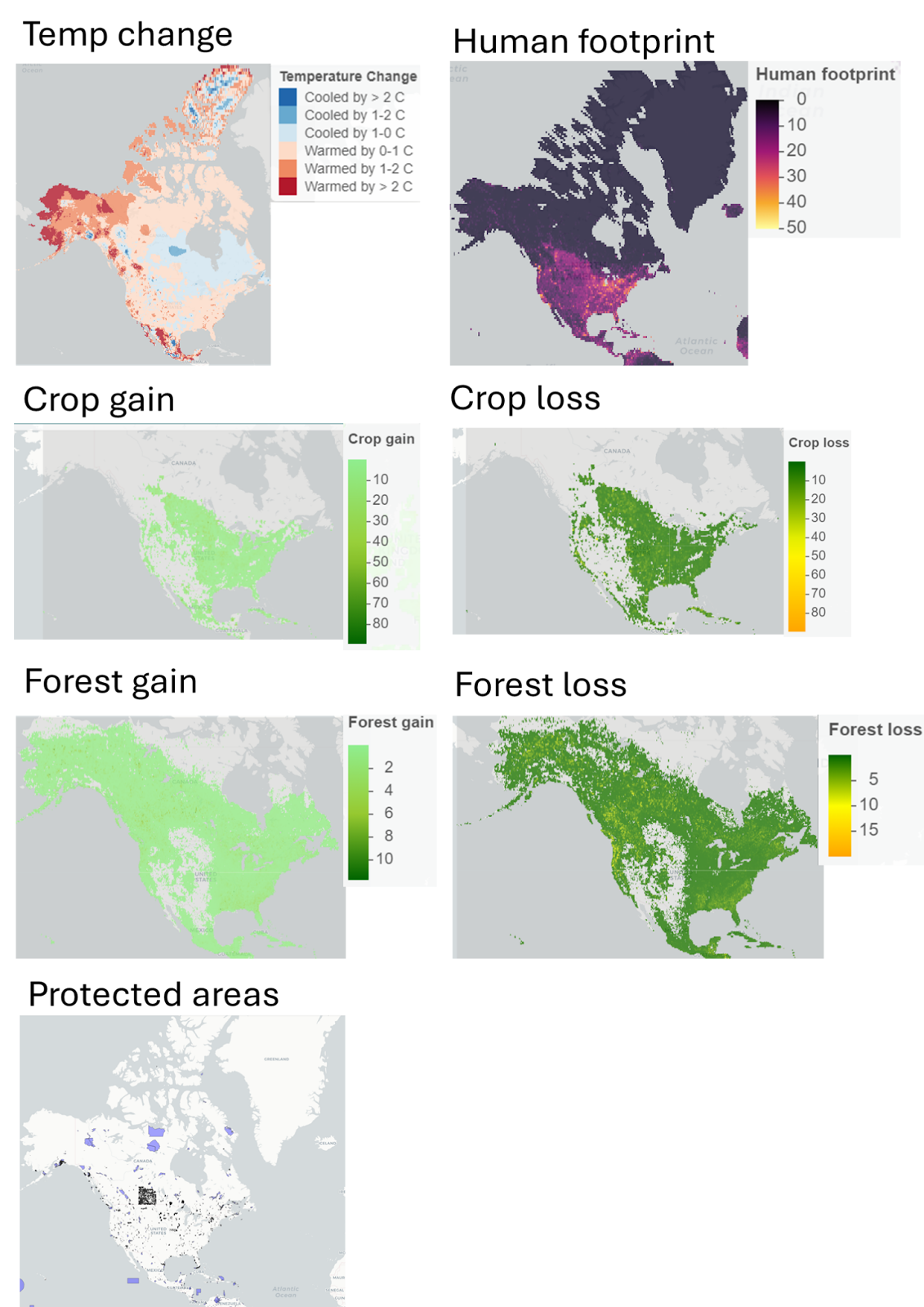
**

**Fig. S2**. Spatial data that we used to test our hypotheses about where extra-range observations occur. Note that we categorized the temperature change rasters for visualization purposes but the data used in our models was continuous. The crop rasters are measured in percent while the forest rasters are measured in meters gained/lost. Map lines delineate study areas and do not necessarily depict accepted national boundaries.

**
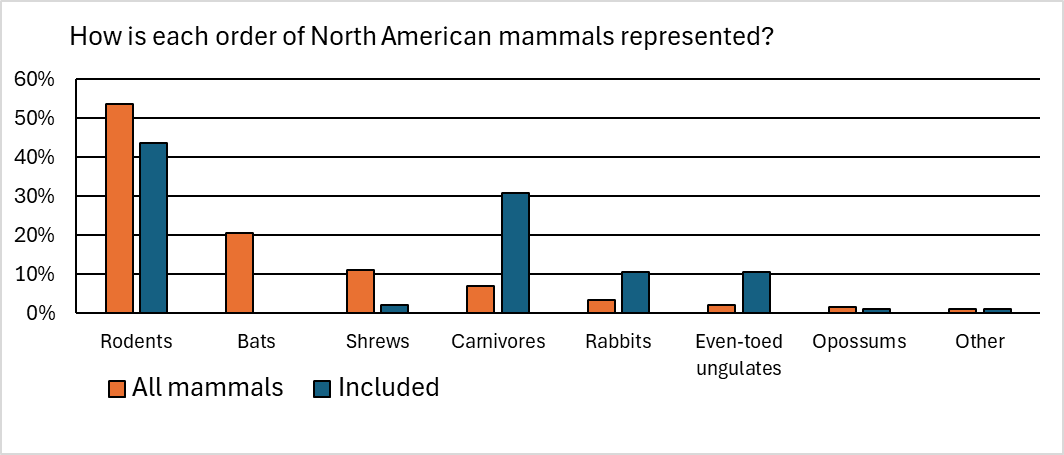
**

**Fig. S3**. The representation of all 774 North American mammals compared to the 94 species for which we investigated extra-range observations for. Bats and shrews are underrepresented in our data, while carnivores, rabbits, and even-toed ungulates are overrepresented.

**Data S1.** Species-specific model estimates for where extra-range observations likely representing natural range expansions occur. We also include a map for each species showing intra-range observations, and extra-range observations colored by whether or not they are a) likely to represent natural range expansions, b) likely to represent introductions or vagrant individuals, or c) are flagged in iNaturalist as introduced but otherwise would have been classified as a natural range expansion.

Link to results: https://figshare.com/s/e154a5dbaf79ffc7e42d

**Table S1.** Summary statistics for each of the 83 species we included in objective one. The proportion columns are the proportion of observations outside IUCN or AOH (area of habitat) ranges.

| **Scientific name** | **Common name** | **Total iNat** | **iNat outside IUCN** | **IUCN proportion** | **iNat outside AOH** | **AOH proportion** | **IUCN range year** |
| --- | --- | --- | --- | --- | --- | --- | --- |
| Alces alces | Moose | 8885 | 2653 | 29.86 | 3653 | 41.11 | 2016 |
| Antilocapra americana | Pronghorn | 4691 | 1848 | 39.39 | 2033 | 43.34 | 2008 |
| Aplodontia rufa | Mountain beaver | 50 | 5 | 10.00 | 6 | 12.00 | 2008 |
| Bassariscus astutus | Ringtail | 1720 | 44 | 2.56 | 97 | 5.64 | 2008 |
| Callospermophilus lateralis | Golden-mantled ground squirrel | 5666 | 70 | 1.24 | 1679 | 29.63 | 2008 |
| Callospermophilus saturatus | Cascade golden-mantled ground squirrel | 613 | 2 | 0.33 | 7 | 1.14 | 2016 |
| Canis lupus | Gray wolf | 242 | 44 | 18.18 | 55 | 22.73 | 2018 |
| Castor canadensis | American beaver | 20150 | 873 | 4.33 | 4350 | 21.59 | 2016 |
| Cervus canadensis | Wapiti | 6937 | 1429 | 20.60 | 1660 | 23.93 | 2018 |
| Condylura cristata | Star-nosed mole | 727 | 29 | 3.99 | 117 | 16.09 | 2008 |
| Conepatus leuconotus | American hog-nosed skunk | 233 | 10 | 4.29 | 58 | 24.89 | 2016 |
| Dasypus novemcinctus | Nine-banded armadillo | 10708 | 460 | 4.30 | 741 | 6.92 | 2014 |
| Didelphis virginiana | Virginia opossum | 18088 | 1055 | 5.83 | 3846 | 21.26 | 2008 |
| Erethizon dorsatum | North American porcupine | 7923 | 1327 | 16.75 | 1715 | 21.65 | 2016 |
| Geomys pinetis | Southeastern pocket gopher | 164 | 14 | 8.54 | 18 | 10.98 | 2008 |
| Gulo gulo | Wolverine | 9 | 2 | 22.22 | 6 | 66.67 | 2016 |
| Herpailurus yagouaroundi | Jaguarundi | 163 | 14 | 8.59 | 24 | 14.72 | 2015 |
| Ictidomys tridecemlineatus | Thirteen-lined ground squirrel | 1745 | 168 | 9.63 | 637 | 36.50 | 2008 |
| Leopardus pardalis | Ocelot | 547 | 30 | 5.48 | 90 | 16.45 | 2015 |
| Lepus alleni | Antelope jackrabbit | 306 | 4 | 1.31 | 106 | 34.64 | 2019 |
| Lepus americanus | Snowshoe hare | 4404 | 255 | 5.79 | 696 | 15.80 | 2019 |
| Lepus arcticus | Arctic hare | 82 | 52 | 63.41 | 43 | 52.44 | 2019 |
| Lepus californicus | Black-tailed jackrabbit | 6593 | 242 | 3.67 | 703 | 10.66 | 2019 |
| Lepus townsendii | White-tailed jackrabbit | 1884 | 3 | 0.16 | 923 | 48.99 | 2008 |
| Lontra canadensis | North American river otter | 7738 | 1697 | 21.93 | 6475 | 83.68 | 2015 |
| Lynx canadensis | Canada lynx | 340 | 21 | 6.18 | 36 | 10.59 | 2016 |
| Lynx rufus | Bobcat | 8900 | 431 | 4.84 | 1295 | 14.55 | 2016 |
| Marmota caligata | Hoary marmot | 1496 | 21 | 1.40 | 371 | 24.80 | 2018 |
| Marmota flaviventris | Yellow-bellied marmot | 5143 | 1502 | 29.20 | 3090 | 60.08 | 2008 |
| Marmota monax | Groundhog | 15320 | 594 | 3.88 | 2120 | 13.84 | 2016 |
| Marmota olympus | Olympic marmot | 143 | 6 | 4.20 | 6 | 4.20 | 2008 |
| Marmota vancouverensis | Vancouver Island marmot | 34 | 9 | 26.47 | 17 | 50.00 | 2017 |
| Martes americana | American marten | 486 | 91 | 18.72 | 110 | 22.63 | 2016 |
| Mustela nivalis | Least weasel | 41 | 3 | 7.32 | 5 | 12.20 | 2016 |
| Myocastor coypus | Nutria | 3145 | 3145 | 100.00 | 3145 | 100.00 | 2008 |
| Nasua narica | White-nosed coati | 5806 | 200 | 3.44 | 1232 | 21.22 | 2016 |
| Neofiber alleni | Round-tailed muskrat | 8 | 0 | 0.00 | 3 | 37.50 | 2011 |
| Neogale frenata | Long-tailed weasel | 1472 | 58 | 3.94 | 934 | 63.45 | 2016 |
| Neogale vison | American mink | 5313 | 805 | 15.15 | 1700 | 32.00 | 2016 |
| Neotamias canipes | Gray-footed chipmunk | 219 | 1 | 0.46 | 7 | 3.20 | 2016 |
| Neotamias cinereicollis | Gray-collared chipmunk | 37 | 9 | 24.32 | 12 | 32.43 | 2008 |
| Neotamias dorsalis | Cliff chipmunk | 655 | 107 | 16.34 | 139 | 21.22 | 2008 |
| Neotoma cinerea | Bushy-tailed woodrat | 128 | 4 | 3.13 | 42 | 32.81 | 2008 |
| Neurotrichus gibbsii | American shrew mole | 517 | 40 | 7.74 | 47 | 9.09 | 2008 |
| Ochotona collaris | Collared pika | 125 | 16 | 12.80 | 88 | 70.40 | 2016 |
| Ochotona princeps | American pika | 3052 | 19 | 0.62 | 2017 | 66.09 | 2016 |
| Ondatra zibethicus | Muskrat | 12797 | 803 | 6.27 | 10991 | 85.89 | 2016 |
| Oreamnos americanus | Mountain goat | 1257 | 652 | 51.87 | 800 | 63.64 | 2022 |
| Oryzomys couesi | Coues' rice rat | 9 | 0 | 0.00 | 5 | 55.56 | 2008 |
| Oryzomys palustris | Marsh rice rat | 28 | 2 | 7.14 | 14 | 50.00 | 2008 |
| Otospermophilus beecheyi | California ground squirrel | 18392 | 2403 | 13.07 | 13128 | 71.38 | 2008 |
| Otospermophilus variegatus | Rock squirrel | 7103 | 496 | 6.98 | 2363 | 33.27 | 2008 |
| Ovibos moschatus | Muskox | 190 | 44 | 23.16 | 165 | 86.84 | 2022 |
| Ovis canadensis | Bighorn sheep | 2319 | 1490 | 64.25 | 1727 | 74.47 | 2008 |
| Ovis dalli | Dall sheep | 99 | 36 | 36.36 | 43 | 43.43 | 2008 |
| Pecari tajacu | Collared peccary | 4372 | 505 | 11.55 | 810 | 18.53 | 2008 |
| Pekania pennanti | Fisher | 1142 | 96 | 8.41 | 200 | 17.51 | 2016 |
| Poliocitellus franklinii | Franklin's ground squirrel | 468 | 113 | 24.15 | 213 | 45.51 | 2008 |
| Procyon lotor | Raccoon | 40527 | 3035 | 7.49 | 9359 | 23.09 | 2015 |
| Puma concolor | Puma | 1717 | 53 | 3.09 | 106 | 6.17 | 2015 |
| Rangifer tarandus | Caribou | 306 | 93 | 30.39 | 166 | 54.25 | 2016 |
| Sciurus aberti | Abert's squirrel | 1111 | 455 | 40.95 | 509 | 45.81 | 2008 |
| Sciurus arizonensis | Arizona gray squirrel | 650 | 31 | 4.77 | 44 | 6.77 | 2016 |
| Sciurus carolinensis | Eastern gray squirrel | 100508 | 10996 | 10.94 | 25598 | 25.47 | 2008 |
| Sciurus griseus | Western gray squirrel | 4626 | 964 | 20.84 | 1883 | 40.70 | 2008 |
| Sciurus nayaritensis | Mexican fox squirrel | 132 | 49 | 37.12 | 78 | 59.09 | 2008 |
| Sciurus niger | Eastern fox squirrel | 48897 | 19074 | 39.01 | 35230 | 72.05 | 2008 |
| Spilogale gracilis | Western spotted skunk | 280 | 26 | 9.29 | 35 | 12.50 | 2016 |
| Sus scrofa | Wild pig | 3450 | 3450 | 100.00 | 3450 | 100.00 | 2008 |
| Sylvilagus idahoensis | Pygmy rabbit | 7 | 2 | 28.57 | 4 | 57.14 | 2016 |
| Sylvilagus palustris | Marsh rabbit | 2570 | 363 | 14.12 | 690 | 26.85 | 2019 |
| Tamias striatus | Eastern chipmunk | 28525 | 861 | 3.02 | 4954 | 17.37 | 2008 |
| Tamiasciurus douglasii | Douglas squirrel | 4171 | 515 | 12.35 | 840 | 20.14 | 2016 |
| Tamiasciurus hudsonicus | Red squirrel | 23928 | 3550 | 14.84 | 7796 | 32.58 | 2009 |
| Taxidea taxus | American badger | 1336 | 15 | 1.12 | 45 | 3.37 | 2008 |
| Urocitellus parryii | Arctic ground squirrel | 601 | 23 | 3.83 | 246 | 40.93 | 2012 |
| Urocyon cinereoargenteus | Gray fox | 9003 | 157 | 1.74 | 775 | 8.61 | 2016 |
| Urocyon littoralis | Island fox | 183 | 6 | 3.28 | 20 | 10.93 | 2013 |
| Ursus americanus | American black bear | 12949 | 1434 | 11.07 | 1966 | 15.18 | 2016 |
| Ursus arctos | Brown bear | 723 | 125 | 17.29 | 165 | 22.82 | 2017 |
| Ursus maritimus | Polar bear | 212 | 0 | 0.00 | 107 | 50.47 | 2012 |
| Vulpes lagopus | Arctic fox | 109 | 19 | 17.43 | 77 | 70.64 | 2014 |
| Vulpes vulpes | Red fox | 16118 | 1406 | 8.72 | 16118 | 100.00 | 2016 |
